# Reversible integer wavelet transform for blind image hiding method

**DOI:** 10.1371/journal.pone.0176979

**Published:** 2017-05-12

**Authors:** Nazeer Muhammad, Nargis Bibi, Zahid Mahmood, Tallha Akram, Syed Rameez Naqvi

**Affiliations:** 1 Department of Mathematics, COMSATS Institute of Information Technology, Wah Cantt., Pakistan; 2 Department of Computer Science, Fatima Jinnah Women University, Rawalpindi, Pakistan; 3 Department of Electrical Engineering, COMSATS Institute of Information Technology, Abbotabad, Pakistan; 4 Department of Electrical Engineering, COMSATS Institute of Information Technology, Wah Cantt., Pakistan; Nanjing Normal University, CHINA

## Abstract

In this article, a blind data hiding reversible methodology to embed the secret data for hiding purpose into cover image is proposed. The key advantage of this research work is to resolve the privacy and secrecy issues raised during the data transmission over the internet. Firstly, data is decomposed into sub-bands using the integer wavelets. For decomposition, the Fresnelet transform is utilized which encrypts the secret data by choosing a unique key parameter to construct a dummy pattern. The dummy pattern is then embedded into an approximated sub-band of the cover image. Our proposed method reveals high-capacity and great imperceptibility of the secret embedded data. With the utilization of family of integer wavelets, the proposed novel approach becomes more efficient for hiding and retrieving process. It retrieved the secret hidden data from the embedded data blindly, without the requirement of original cover image.

## Introduction

Blind data hiding is a vibrant issue of Internet communications which is frequently used for sending, receiving, or storing the secret information [1]. As the number of secrets retained via internet are upsurged, the demand for the fortification from frequent embezzlement is increased dramatically, which results in the fictitious propaganda. The images are susceptible to imperceptible alterations by the common user observations. An effective way to overcome the unpredictability is to encrypt the meaningful secret data into a meaningless dummy data. The subsequent dummy data can be implicit by those individuals only who know the distinct key parameters for yielding to candid form with complete sequence of accurate algorithm. Secret encryption permits secure authentications and data confidentiality. For secret digital information handling, it is significant to hide secret digital message into some cover image so that it does not disclose unique subjects [2].

Digital data hiding methods can be considered as reversible, irreversible, blind, or non-blind, respectively [2]. Reversible methods are used to restore the original cover image from embedded data by retrieving the hidden secrets. In irreversible technique, once the secret data is concealed into the original cover image, it results in the lost of original cover which cannot be recovered at the retrieving stages from the embedded media [3]. In military and medical image processing, images are collected at an expensive price and are repeatedly subjected to additional treating, such as massive magnification and interpolation [4]. Any alteration may hamper analysis of the digital image [5]. Another benefit of the reversible data hiding is that access to unique contents of the expensive images can be controlled.

In the blind data hiding method, embedding the secret data can be detected from the embedded cover image without admittance of the original cover image. However, an original cover image is required during detection phase of the non-blind data hiding method. However, the blind detection has wider applicability because ease of the original cover data is not always available at retrieving end. However, it is not realistic assumption to use the common image as a cover data, since the cover image and the hidden data of the secret image cannot be surely detached from the communicated data at the detecting end [6].

It has been determined that two key aspects are affecting the secret data hiding process: *(1)* The amount of the hiding capacity (or payload) and the visual eminence of the embedded images [7]. The first factor, a secret data hiding method with slight data adjustment is more protected than that with considerable (high) adjustment since it does not increase any inconsistency of modified cover data. *(2)* Hiding capacity of cover data with high payload is desired because a large amount of private and personal data can be capably conveyed for a number of scenarios, such as intelligence agencies, defense, military, and health organizations [8]. The proposed method’s significant contributions are as follows:

- This paper focuses on the reversible blind data hiding method to increase the privacy and security by assimilating both cryptography and steganography with efficient hiding and detecting processes for the large secret data.
- The proposed technique utilizes Fresnelet transform to achieve a meaningless dummy data of the meaningful secret image data [9]. Consequently, copyrights protection of secret hiding data contents may be performed using the Fresnelet transform [10]. The application of the Fresnelet transform may be acclaimed for multifaceted encryption processes to deliver more reliability and security. Moreover, using the Fresnelet transform, secret data detected from an embedded image can be acquired with high resolution [11].
- Fresnelet transform basis are yielding robust parameters to attain a multi-scale spreading of secret hiding data to establish the key features of the proposed method for privacy and security. It is not possible to achieve the hidden secret message secret hiding data deprived of the accurate key parameters.
- The proposed method considers the integer wavelet transform (*WT*) reversibility property. The secret hiding data can be embedded into a resized approximated cover image which is produced from the input original image using *WT* transform [12]. So, the approximated image can be reversibly reinstated from an embedded data and the secret hiding data.

The rest of this paper is organized as follows. The nomenclature of important expression is stated in Table 1. The Fresnelet transform based encryption is explained in the next section. The subsequent section describes the reversible blind data hiding method. Then the detailed results evaluation is presented. In the last section the conclusions are drawn.

**Table 1:** Nomenclature.

Expression	Explanation
${\tilde{f}}_{\tau }$	Fresnel transform
*ϕ*	Scaling function
*ψ*	Wavelet function
*λ*	Wavelength parameter
*d*	Distance parameter
*WT*	Integer wavelet transform
*F*_*τ*_	Fresnelet transform

## Data encryption process

An approximation model of the diffraction phenomena is developed using the convolution integral ${\tilde{f}}_{\tau }$ through the complex waves propagation [13–15]. The ${\tilde{f}}_{\tau }$ with 1-D case can be expressed for a function $f\in L_{2}\left(R\right)$ as the follows:

$$\begin{array}{c} {\tilde{f}}_{\tau }\left(x\right)=\left(f*k_{\tau }\right)\left(x\right)\;\text{with}\;k_{\tau }\left(x\right)=\frac{1}{\tau }exp\left(i\pi \frac{x^{2}}{\tau^{2}}\right). \end{array}$$ (1)

where *τ* > 0 is the normalizing parameter depending on the wavelength *λ* and the distance *d* as follow:

$$\begin{array}{c} \tau =\sqrt{\lambda d}. \end{array}$$ (2)

The ${\tilde{f}}_{\tau }$ is applied to a wavelet basis [16] and new bases for multi-resolution analysis are obtained [11]. The new bases have been used for digital holograms reconstruction with important parameters: propagating distance, resolution scale, and wavelength, respectively, between image and object plane [11]. The ${\tilde{f}}_{\tau }$ with 2-D case is achieved by using the tensor product in terms 1-D kernel *k*_*τ*_(*x*). Therefore, for $f\in L_{2}\left(R^{2}\right)$,

$$\begin{array}{c} {\tilde{f}}_{\tau }\left(x,y\right)=\left(f*K_{\tau }\right)\left(x,y\right)\;\text{with}\;K_{\tau }\left(x,y\right)=k_{\tau }\left(x\right)k_{\tau }\left(y\right) \end{array}$$ (3)

Since the kernel *K*_*τ*_(*x*, *y*) is separable, the ${\tilde{f}}_{\tau }$ with 1-D is freely expanded to 2-D case. Various useful properties of the ${\tilde{f}}_{\tau }$ can be explained [11]. Among the prominent ones is the unitary property [11]. It facilitates the ${\tilde{f}}_{\tau }$ to perfect reconstruction of a given data *f* as follows:

$$\begin{array}{c} f=\left({\tilde{f}}_{\tau }*K_{\tau }^{-1}\right)\left(x,y\right). \end{array}$$ (4)

The wavelet of 1-D case can be also applied though separable extension to obtain the wavelet of 2-D case [17]. The wavelet transform generated by wavelets with two parameter family ${\left\{\psi_{j,l}\right\}}_{j,\;l\in Z}$ is applied on $L_{2}\left(R\right)$ as convolution integrals, which also defines the Riesz basis for $L_{2}\left(R\right)$, where

$$\begin{array}{c} {\left\{\psi_{j,l}\left(x\right)=2^{j/2}\psi \left(2^{j}x-l\right)\right\}}_{j,\;l\in Z}. \end{array}$$ (5)

The Haar wavelet that produces an orthonormal basis for $L_{2}\left(R\right)$ is considered as a simplest form of a wavelet. This wavelet is used to achieve the perfect reconstruction and multi-resolution analysis of the data as well [18]. The detail features of the wavelet transforms are well explained in [19]. The basis of wavelet transform is used to define the Fresnelet basis with the ${\tilde{f}}_{\tau }$, as follows:

$$\begin{array}{c} {\left\{{\left(\psi_{j,l}\right)}_{\tau }^{∼}\right\}}_{j,\;l\in Z}\;\text{with}\;{\left(\psi_{j,l}\right)}_{\tau }^{∼}\left(x\right)=2^{j/2}{\tilde{\psi }}_{2^{j}\tau }\left(2^{j}x-l\right). \end{array}$$ (6)

With an orthogonal wavelet basis ${\left\{\psi_{j,l}\right\}}_{j,\;l\in Z}$, an orthonormal Fresnelet basis are generated. In this regards, for the fixed value of *τ*, by allowing $\Theta_{j,l}\left(x\right)={\left(\psi_{j,l}\right)}_{\tau }^{∼}\left(x\right)$, we have attained the following Fresnelet decomposition:

$$\begin{array}{c} f=\sum_{j,l}\;c_{j,l}\;\Theta_{j,l}\;\text{with}\;\;\;c_{j,l}=\left\langle f,\Theta_{j,l}\right\rangle . \end{array}$$ (7)

The coefficients *c*_*j*,*l*_ in Eq (5) are known as the Fresnelet coefficients. Using the separable nature of the Fresnelet transform, the Fresnelet transform with 1-D case can be extended to 2-D case. In this way, the tensor product with four combinations $\gamma_{\tau }^{\left(ll\right)},\;\;\gamma_{\tau }^{\left(lh\right)},\;\;\gamma_{\tau }^{\left(hl\right)},\;\;\text{and}\;\gamma_{\tau }^{\left(hh\right)},$ for establishing the approximate, horizontal, vertical, and diagonal sub-bands, respectively, as follow:

$$\begin{array}{c} \gamma_{\tau }^{\left(ll\right)}={\left(\phi_{j,l}\right)}_{\tau }^{∼}\left(x\right){\left(\phi_{j,l}\right)}_{\tau }^{∼}\left(y\right), \end{array}$$ (8)

$$\begin{array}{c} \gamma_{\tau }^{\left(lh\right)}={\left(\phi_{j,l}\right)}_{\tau }^{∼}\left(x\right){\left(\psi_{j,l}\right)}_{\tau }^{∼}\left(y\right), \end{array}$$ (9)

$$\begin{array}{c} \gamma_{\tau }^{\left(hl\right)}={\left(\psi_{j,l}\right)}_{\tau }^{∼}\left(x\right){\left(\phi_{j,l}\right)}_{\tau }^{∼}\left(y\right), \end{array}$$ (10)

$$\begin{array}{c} \gamma_{\tau }^{\left(hh\right)}={\left(\psi_{j,l}\right)}_{\tau }^{∼}\left(x\right){\left(\psi_{j,l}\right)}_{\tau }^{∼}\left(y\right), \end{array}$$ (11)

where a low-pass filter is generated by the Eq (8) and high-pass filters are generated by Eqs (9)–(11). Four types of the Fresnelet coefficients are achieved by applying the basis functions above to data *f*:

$$\begin{array}{c} f_{\tau ,d}^{\left(ll\right)}=\left\langle f,\gamma_{\tau }^{\left(ll\right)}\right\rangle , \end{array}$$

$$\begin{array}{c} f_{\tau ,d}^{\left(lh\right)}=\left\langle f,{\;\gamma }_{\tau }^{\left(lh\right)}\right\rangle , \end{array}$$

$$\begin{array}{c} f_{\tau ,d}^{\left(hl\right)}=\left\langle f,\gamma_{\tau }^{\left(hl\right)}\right\rangle , \end{array}$$

$$\begin{array}{c} f_{\tau ,d}^{\left(hh\right)}=\left\langle f,\gamma_{\tau }^{\left(hh\right)}\right\rangle . \end{array}$$

**Fig 1:**
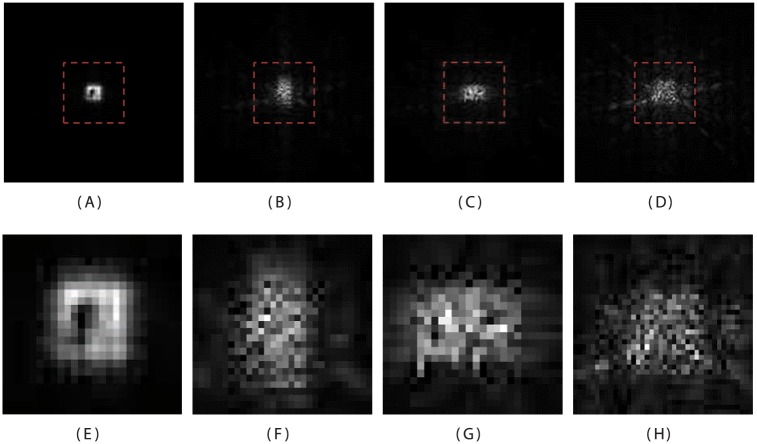
Fresnelet transformed decomposition of the secret data of Bridge image with key parameter *d*_1_ = 1*m*. (A) approximation data, (B) the horizontal detail data, (C) the vertical detail data, (D) the diagonal detail data, (E) zoomed-in view of approximation data, (F) zoomed-in view of horizontal detail data, (G) zoomed-in view of vertical detail data, and (H) zoomed-in view of diagonal detail data.

**Fig 2:**
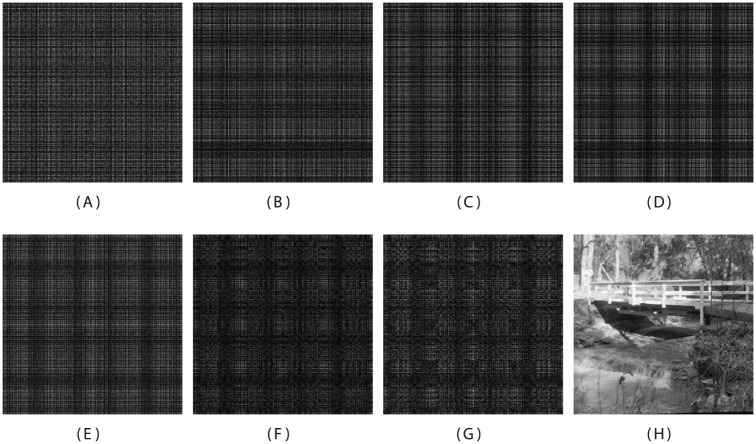
Fresnelet transformed encryption application. (A) The corresponding approximation data obtained from the Fresnelet transformed application on the Fig 1A with key parameter *d*_2_ = 10^−4^ *m*, (B) the corresponding horizontal detail data obtained from the Fresnelet transformed application on the Fig 1B, (C) the corresponding vertical detail data obtained from the Fresnelet transformed application on the Fig 1C, (D) the corresponding diagonal detail data obtained from the Fresnelet transformed application on the Fig 1D, (E) the magnitude of complex valued scrambled data, (F) the real part of the scrambled data, (G) the imaginary part of the scrambled data, (H) the reconstructed image using the inverse Fresnelet transform process from the real part and the imaginary part of the scrambled data.

The coefficient $f_{\tau ,d}^{\left(ll\right)}$ represents the low-passed data and the coefficients $f_{\tau ,d}^{\left(lh\right)}$, $f_{\tau ,d}^{\left(hl\right)}$, and $f_{\tau ,d}^{\left(hh\right)}$, are high-passed detail data. The Fresnelet coefficients of the secret data are revealed in Fig 1. Instead of the simple ${\tilde{f}}_{\tau }$ (typically derived for digital off-axis hologram reconstruction) [12], it is worth revealing in Fig 1, that the forward Fresnelet transform can be used to process the meaningful information into dummy (encrypted) data with four different sub-bands. This data is totally encrypted. These four sub-bands in the set-up of complex data are shown in Fig 2. The inverse of the Fresnelet coefficients $f_{\tau ,d}^{\left(ll\right)}$, $f_{\tau ,d}^{\left(lh\right)}$, $f_{\tau ,d}^{\left(hl\right)}$, and $f_{\tau ,d}^{\left(hh\right)}$ can be obtained using the tensor product as follows:

$$\begin{array}{c} {\hat{f}}_{\tau ,d}^{\left(ll\right)}=\left\langle f_{\tau ,d}^{\left(ll\right)},\gamma_{\tau }^{-(ll)}\right\rangle , \end{array}$$

$$\begin{array}{c} {\hat{f}}_{\tau ,d}^{\left(lh\right)}=\left\langle f_{\tau ,d}^{\left(lh\right)},{\;\gamma }_{\tau }^{-(lh)}\right\rangle , \end{array}$$

$$\begin{array}{c} {\hat{f}}_{\tau ,d}^{\left(hl\right)}=\left\langle f_{\tau ,d}^{\left(hl\right)},\gamma_{\tau }^{-(hl)}\right\rangle , \end{array}$$

$$\begin{array}{c} {\hat{f}}_{\tau ,d}^{\left(hh\right)}=\left\langle f_{\tau ,d}^{\left(hh\right)},\gamma_{\tau }^{-(hh)}\right\rangle . \end{array}$$

Where inverse Fresnelet transform, is also equivalent to inverse of integer wavelet transform. This provide the original function *f* using the following expression.

$$\begin{array}{c} f=\sum_{j=0}\;\lfloor {\hat{f}}_{\tau ,d}^{\left(ll\right)},{\hat{f}}_{\tau ,d}^{\left(lh\right)},{\hat{f}}_{\tau ,d}^{\left(hl\right)},{\hat{f}}_{\tau ,d}^{\left(hh\right)}\rfloor , \end{array}$$ (12)

where *j* = 0, represents the reconstruction scale same as before decomposition process: propagating waves comes back to their original position. The magnitudes of secret data of the Bridge image is obtained using the Fresnelet coefficients as shown in Fig 2. Note that a reconstruction of a secret data using the unitary property of the Fresnelet transformation is attained using the forward Fresnelet transform. The reconstruction results have complex data. In Fig 1, the first row shows the coded secret data of Bridge image. Moreover, input image, Lena, Peppers, Boat, Airplane, and Mandrill are considered as a cover information. These are obtained from standard bench mark images of SIPI image database (University of Southern California). The Bridge image is propagated using the Fresnelet transform with the distance parameter *d*_1_ = 1*m*. The noticeable areas with dotted lines of the corresponding images in the first row are representing in the second row (zoomed-in views). First figure in second row of Fig 2 is demonstrating the inverse propagation of the corresponding sub-band data of the first row

## Data hiding process

In the proposed method, we apply the *WT* for decomposing and reconstructing the cover image. The lifting framework [20] is used to perform the integer wavelet transform that has low storage cost without extensive complexity of computation. In numerical simulation, the integer Haar wavelet transform is used for decomposing and reconstruction of the cover image. The data hiding proposed method is comprised on two steps: embedding process and retrieving process.

### The embedding process

The two phases are involved in the embedding process. In the first, the encryption of the secret data is performed and in the second, the cover image is decomposed in which the encryption from the secret data is embedded. To preserve confidential information, secret image is decomposed based on the Haar wavelet using the Fresnelet transform. At first, the secret data *f* is transformed using the *F*_*τ*_ with the initial distance parameter key, *d*_1_ = 1*m*, as follow:

$$\begin{array}{c} F_{\tau }\left(f,d_{1}\right)=\left(\begin{array}{cc} f_{\tau ,d_{1}}^{\left(ll\right)} & f_{\tau ,d_{1}}^{\left(hl\right)}\\ f_{\tau ,d_{1}}^{\left(lh\right)} & f_{\tau ,d_{1}}^{\left(hh\right)} \end{array}\right). \end{array}$$ (13)

At second, a scrambled data *D* is generated from the decomposed data of *f* via the inverse Fresnelet transform *IF*_*τ*_ on using the subsequent distance parameter key, *d*_2_ = 10^−4^ *m*, as follow:

$$\begin{array}{c} D={IF}_{\tau }\left\{\left(\begin{array}{cc} f_{\tau ,d_{1}}^{\left(ll\right)} & f_{\tau ,d_{1}}^{\left(hl\right)}\\ f_{\tau ,d_{1}}^{\left(lh\right)} & f_{\tau ,d_{1}}^{\left(hh\right)} \end{array}\right),{\;d}_{2}\right\}. \end{array}$$

The encrypted data is obtained from the Bridge secret data as shown in Fig 2. We use the Bridge secret data as an information image which is taken from SIPI image database, University of Southern California: http://sipi.usc.edu/database/database.php (Volume 3: Miscellaneous). The nature of the encrypted data are complex valued because of the Fresnelet transform application [12]. This complex data can be separated into the real part and the imaginary part: *D*_*re*_ and *D*_*im*_. The embedding of those parts are made into suitable sub-band (detail parts) of the decomposed cover image as shown in Eqs (15) and (16). The integer wavelet transform is employed on a cover image *S*, for obtaining the sub-bands data in which the encrypted secret data for hiding purpose in detail parts will be embedded. Prior to use a cover image *S*, we consider an input image *C* (standard benchmark images). The one level decomposition is performed on *C* At the coarser resolution level *j*-1 (where *j* be the finest resolution level), four sub-band images are attained as follows:

$$\begin{array}{c} WT\left(C\right)=\left(\begin{array}{cc} C_{j-1}^{\left(ll\right)} & C_{j-1}^{\left(hl\right)}\\ C_{j-1}^{\left(lh\right)} & C_{j-1}^{\left(hh\right)} \end{array}\right). \end{array}$$

The *C* is transformed to an approximated data $C_{j-1}^{\left(ll\right)}$ on employing the low-pass wavelet filter along its rows and columns. The *C* is transformed to a horizontally oriented detail data $C_{j-1}^{\left(lh\right)}$ on employing the low-pass wavelet filter along its rows and high-pass wavelet filter along its columns. The *C* is transformed to a vertically oriented detail data $C_{j-1}^{\left(hl\right)}$ on employing the high-pass wavelet filter along its rows and low-pass wavelet filter along its columns. Similarly, the *C* is transformed to a detailed data $C_{j-1}^{\left(hh\right)}$ on employing the high-pass wavelet filter along its rows and columns. Notice that the low-pass sub-band approximated data $C_{j-1}^{\left(ll\right)}$ is containing high energy. The approximated data $C_{j-1}^{\left(ll\right)}$ obtained using the *WT* is considered as an original image *I* in our proposed method and all high-passed details $C_{j-1}^{\left(hl\right)}$, $C_{j-1}^{\left(lh\right)}$, and $C_{j-1}^{\left(hh\right)}$ are discarded. We scale *I* twice to its size using bi-cubic interpolation and obtain the resized data *S*. This resized data *S* is considered as the cover image and decomposed into four sub-bands using the *WT*.

$$\begin{array}{c} WT\left(S\right)=\left(\begin{array}{cc} S_{j-1}^{\left(ll\right)} & S_{j-1}^{\left(hl\right)}\\ S_{j-1}^{\left(lh\right)} & S_{j-1}^{\left(hh\right)} \end{array}\right). \end{array}$$

The decomposition of the *S* is providing us the sub-band data: the low-passed data $S_{j-1}^{\left(ll\right)}$, the horizontal detail data $S_{j-1}^{\left(lh\right)}$, the vertical detail data $S_{j-1}^{\left(hl\right)}$, and the diagonal detail data $S_{j-1}^{\left(hh\right)}$, respectively. The detail sub-band data coefficients are corresponding to edges, corners, and textures, respectively. These are robust in nature and easily can adjust the secret data for embedding purpose [3]. Moreover, in most of the images, the high frequency coefficients of *WT* follow Laplacian-like distribution are suitable for data hiding [21]. So, we embed the encrypted secret data into the $S_{j-1}^{\left(hl\right)}$ and $S_{j-1}^{\left(lh\right)}$. The real part *D*_*re*_ of the encrypted data is embedded into the $S_{j-1}^{\left(hl\right)}$, whereas the imaginary part *D*_*im*_ of the encrypted data is embedded into the $S_{j-1}^{\left(lh\right)}$ as Eqs (15) and (16). A scale parameter *α* is introduced that controls the weight of embedding of encrypted secret data as a strength factor as shown in Eq (14). Its values lie between 0 and 1, depend on low-passed data of cover image $S_{j-1}^{\left(ll\right)}$ and normalizing agent Γ. We choose value of Γ = 0.075 following the [18] to produce *α* as follows:

$$\begin{array}{c} \alpha =\frac{\bar{|S_{j-1}^{ll}|}}{max|S_{j-1}^{ll}|}\times \Gamma , \end{array}$$ (14)

$$\begin{array}{c} {\tilde{S_{j-1}^{\left(hl\right)}}\;=S_{j-1}^{\left(hl\right)}\;+\alpha D_{im}}_{\;}, \end{array}$$ (15)

$$\begin{array}{c} {\tilde{S_{j-1}^{\left(lh\right)}}\;=S_{j-1}^{\left(lh\right)}\;+\alpha D_{re}}_{\;}, \end{array}$$ (16)

where $\tilde{S_{j-1}^{\left(hl\right)}}$ and $\tilde{S_{j-1}^{\left(lh\right)}}$ are the adapted detail data containing the encrypted secret data. During the reconstruction process, we use the original image *I* as a replacement for the resized data $S_{j-1}^{\left(ll\right)}$. This is used for attaining high imperceptibility and a reliable retrieving of the encrypted data from the embedded information as discussed in Section.

$$\begin{array}{c} E=IWT(\begin{array}{cc} I & \tilde{S_{j-1}^{\left(hl\right)}}\\ \tilde{S_{j-1}^{\left(lh\right)}} & S_{j-1}^{\left(hh\right)} \end{array}). \end{array}$$ (17)

On embedding the imaginary and the real parts of *D* in the definite details of *S*, an inverse integer wavelet transform (*IWT*) is employed in the above reconstruction process that produces an information embedded image *E* as shown in Eq (17).

### The retrieving process

**Fig 3:**
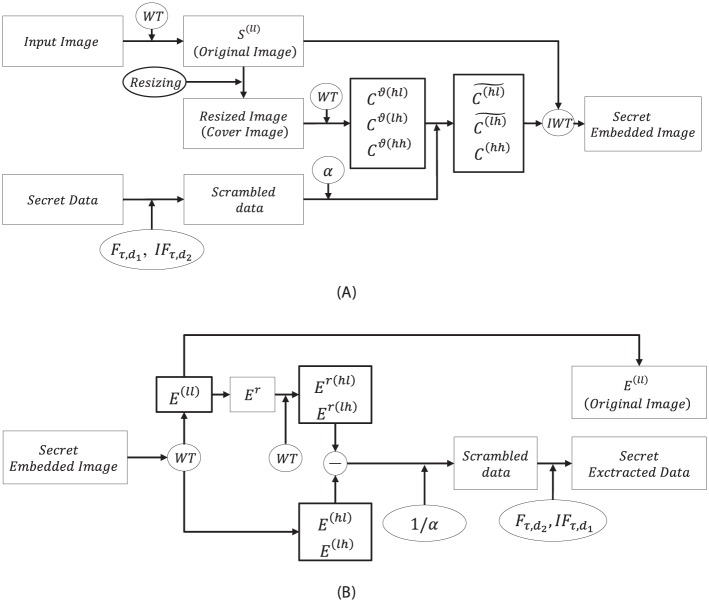
The proposed method processing in flow-chart. (A) the embedding and (B) the retrieving.

The reverse of the embedding process is the retrieving process as shown in Fig 3(B). At first, we decompose the information embedded image *E* into $E_{j-1}^{\left(ll\right)}$, $E_{j-1}^{\left(hl\right)}$, $E_{j-1}^{\left(lh\right)}$, and $E_{j-1}^{\left(hh\right)}$, respectively, on using the *IWT*. Notice that the approximated data $E_{j-1}^{\left(ll\right)}$ is the original image *I* as used in Eq (17). The high frequency sub-band data $E_{j-1}^{\left(hl\right)}$, $E_{j-1}^{\left(lh\right)}$, and $E_{j-1}^{\left(hh\right)}$ are detailed data sets of *E* that are preserved the information in the $E_{j-1}^{\left(hl\right)}$ and $E_{j-1}^{\left(lh\right)}$. Bi-cubic interpolation is used for resizing the low-pass approximated sub-band data $E_{j-1}^{\left(ll\right)}$ with the equal size as that of the *E* to obtain the resized data *E*^*r*^. *E*^*r*^ is decomposed further using the *WT* for retrieving the embedded secret data. Then we get the $E_{j-1}^{r(ll)}$, $E_{j-1}^{r(hl)}$, $\;E_{j-1}^{r(lh)}$, and $E_{j-1}^{r(hh)}$, respectively. The encrypted data (in form of imaginary and real parts) are extracted using the high detail data $E_{j-1}^{r(hl)}$ and $E_{j-1}^{r(lh)}$ by subtracting from the data $E_{j-1}^{\left(hl\right)}$ and $E_{j-1}^{\left(lh\right)}$ of the information embedded data, respectively. Moreover, for obtaining encrypted secret data, the output of the difference data are divided by *α*. The extraction of the encrypted imaginary and real parts are unified in the form of complex data. Furthermore, an inverse Fresnelet transforms is used to this complex encrypted data with the same key parameter in order to provide a meaningful secret data.

### Reversible criteria of the proposed method

In proposed method embedding and retrieving processes are performed in *WT* domain. Recall that the integer wavelet transform is mapped integers to integers. In the lifting scheme, it is particularly useful for supporting the lossless reproduction of an original image [20]-[26]. Note that the lifting scheme for *WT* is realized by its filter banks known as uniformly maximally decimated. These filter banks are carrying out the ladder networks to execute the polyphase filtering [20, 27]. Even in the existence of quantization error such networks can be acted invertible, especially, the rounding error is generated by using the finite-precision arithmetic (see supporting information S1 File)-Appendix. We refer, for instance, to [20] for further details.

The proposed method employs the Haar wavelet filter to the integer sort of the lifting scheme which as follows:

#### Decomposition transform

$$\begin{array}{c} \vartheta_{j+1,i}=\zeta_{j,2i+1}-\zeta_{j,2i}, \end{array}$$

$$\begin{array}{c} \zeta_{j+1,i}=\zeta_{j,2k}+\left\lfloor \frac{\vartheta_{j+1,i}}{2}\right\rfloor . \end{array}$$

#### Reconstruction transform

$$\begin{array}{c} \zeta_{j-1,2i}=\zeta_{j,i}-\left\lfloor \frac{\vartheta_{j,i}}{2}\right\rfloor , \end{array}$$

$$\begin{array}{c} \zeta_{j-1,2i+1}=\vartheta_{j,i}-\zeta_{j-1,2i}. \end{array}$$

where *ζ*_*j*,*i*_ and *ϑ*_*j*,*i*_ are the *i*^*th*^ components of low and high sub band wavelet coefficients at the *j*^*th*^ level, respectively [20]. Also, ⌊•⌋ is a floor operation, which is an integer kind of the linear wavelet transform that holds the rounding error identity.

### Rounding error identity

Where *∀*, *s* ∈ z and *∀*, *t* ∈ z where *t* = *o*, the subsequent identity holds:

$$\begin{array}{c} {}[s/t]=\lfloor (s+t-1)/t\rfloor . \end{array}$$ (18)

#### Proof

*Let* *t* ≠ 0, the function that satisfies the relationships is the mod function as follows:

1. *mod*(*s*, *t*) = *s*, for 0 ≤ *s* ≤ *t* − 1.
2. *mod*(*s* + *t*, *t*) = *mod*(*s*, *t*).

Furthermore, it can be observed from the above mentioned two properties

$$\begin{array}{c} mod(-s,t)=t-1-mod(s-1,t),\;\text{for}\;t\neq 0 \end{array}$$ (19)

where β∈R, the floor function ⌊*β*⌋ denotes the leading integer, and the ceiling function ⌈*β*⌉ represents the least integer. For all s∈Z, it can be shown for the ceiling and floor functions:

$$\begin{array}{c} \lceil \beta \rceil =-\lfloor -\beta \rfloor . \end{array}$$ (20)

From [20] we note 0 ≤ *frac* *β* ≤ 1, where ∀, β∈R. The mod function is defined as

$$\begin{array}{c} mod(s,t)≜s-t\lfloor s/t\rfloor ,\;\text{such that}\;s,t\in Z. \end{array}$$ (21)

where the mod function calculates the nonnegative remainder values such that *s* is divided by *t*. From Eqs (20) and (21), we deduce the following identity

$$\begin{array}{c} \left\lfloor s/t\right\rfloor =\frac{s-mod(s,t)}{t}\;\text{for}\;t\neq 0. \end{array}$$ (22)

$$\begin{array}{c} \left\lceil s/t\right\rceil =\frac{s+mod(-s,t)}{t}\;\text{for}\;t\neq 0. \end{array}$$ (23)

Now we consider the right-hand side Eq (18) to further manipulate Eq (22) as follows:

$$\begin{array}{ccccc} \left\lfloor (s+t-1)/t\right\rfloor & = & \lfloor (s-t)/t\rfloor +1, & = & (s+t-1-mod(s-1,t))/t. \end{array}$$ (24)

From Eqs (21)–(23), we can deduce the following expression:

$$\begin{array}{c} \lfloor (s+t-1)/t\rfloor =(s+mod(-s,t))/t=\lceil s/t\rceil . \end{array}$$ (25)

Therefore, the identity given in Eq (18) holds. The identity given in Eq (24) allows us to implement the ceiling of a quotient which is practically useful in terms of the floor of a quotient and vice versa.

### Overflow and underflow Issue

On embedding the secret image into coefficients of the high frequency using Eqs (15) and (16), it is possible that the 8-bit gray scale values in the entrenched image to some of pixels may go above the upper bound value 255 and/or the lower bound value 0 after inverse *WT* [22]. This is referred to as overflow/underflow. The overflow/underflow problem can be minimized by considering the lossless recovery of an original cover image at the retrieving phase. To prevent this, the common method adopts the histogram modification by creating bookkeeping data [23]. The bookkeeping data technique, however, reduces the capacity of the cover image for embedding secret data as well as increases the computational complexities of the embedding process [24]. The capacity of payload (secret data) in embedded image is defined as the ratio between the numbers of bits (embedded data) to the per pixels of cover image can be expressed as follows:

$$\begin{array}{c} {C\;=\sum_{i=1}^{N}\left\lfloor {log}_{2}\left(q_{i}\right)/\left(M^{2}\right)\right\rfloor }_{\;}, \end{array}$$ (26)

where *q* represents the quantization level *q*_*i*_; *i*, …, *N* and *M*^2^ is the size of cover image where secret data to be embedded. To overcome overflow/underflow issues as well as the capacity and computational complexity issues, the integer wavelet transform method can also be employed. In fact, the invertibility of the lifting scheme for the wavelet transform can resolve those issues [20]. Suppose that the resized cover image *S* is transformed by the *WT* in the embedding process. Consider the embedded image *E* generated by applying the inverse *WT* with high sub-band data $\tilde{C_{j-1}^{\left(hl\right)}}$, $\tilde{C_{j-1}^{\left(lh\right)}}$, and ${\;C}_{j-1}^{\left(hh\right)}$ along with the original image *I* as Eq (17). It can be deduced from Eq (17) that

$$\begin{array}{c} E=IWT(I,\left(C_{j-1}^{\left(hl\right)}+D_{re}\right)+E_{err}^{\left(hl\right)}\text{,}\;\left(C_{j-1}^{\left(lh\right)}\;+D_{im}\right)+E_{err}^{\left(lh\right)}\text{,}\;C_{j-1}^{\left(hh\right)}+E_{err}^{\left(hh\right)}). \end{array}$$ (27)

This shows that underflow/overflow is solely dependent on the errors $E_{err}^{\left(hl\right)}$, $E_{err}^{\left(lh\right)}$, and $E_{err}^{\left(hh\right)}$. These errors are caused by the high sub band data generation and the embedded dummy data *D*_*re*_ and *D*_*im*_. Since *WT* lifting scheme involves in the truncations of $C_{j-1}^{\left(hl\right)}$, $C_{j-1}^{\left(lh\right)}$, and $C_{j-1}^{\left(hh\right)}$ data only during the lifting steps, the rounding off error is unavoidable. So, the rounding off error may be developed in the high sub-band data. However, it does not affect the original image data *I* in Fig 3. Therefore, the reconstruction of an original cover image can be performed without any misrepresentation due to the reversible nature of the lifting scheme for integer wavelet transform.

### Simulation and evaluation

At the first stage, the Peak Signal-to-Noise Ratio (PSNR) a very well-known metric is measured for analyzing the image value of the resized and the embedded images as shown in Table 2.

**Table 2:** Performance comparisons of resizing image (cover image) quality on PSNR (dB) values.

Image	Nearest	Bi-linear	Bi-cubic	Proposed
Lena	28.30	31.41	34.13	**34.75**
Airplane	25.89	29.02	31.28	**31.69**
Peppers	26.79	30.22	31.76	**31.98**
Boat	25.50	27.99	29.95	**30.45**
Mandrill	20.38	22.52	23.63	**24.01**

At later stage, a secret data set with size *N* × *N* is embedded into a cover image of size *M* × *M* with *N* = 256 and *M* = 512. In the proposed method, we consider the Fresnelet transform propagation is performed at: wavelenght *λ* = 632.8 *nm*, a sampling interval size of a CCD plane Δ = 10 *nm* and distances *d*_1_ = 1*m* and *d*_2_ = 10^−4^ *m* [11]. The Fresnelet transform operations with these parameters are employed for the embedding and retrieving phases.

**Fig 4:**
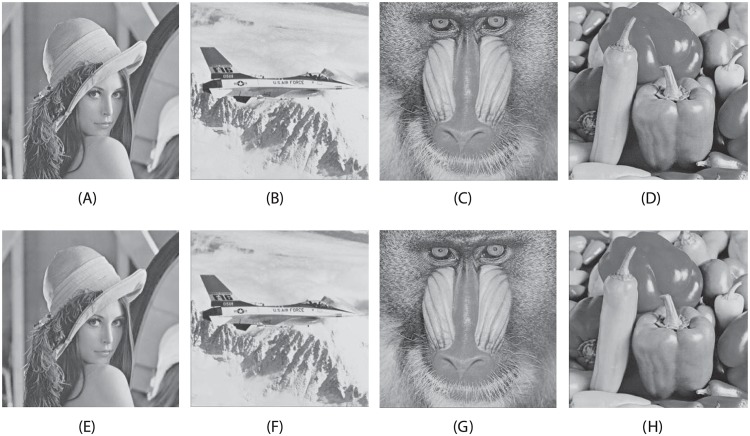
The secret embedded images (carrying secret image Bridge) and recovered input images (lossless recovery after extraction of the secret image). (A) Embedded image Lena (*PSNR* = 33.56), (B) embedded image Airplane (*PSNR* = 31.07), (C) embedded image Mandrill (*PSNR* = 29.97), (D) embedded image Peppers (*PSNR* = 23.89), (E) recovered input image Lena, (F) recovered input image Airplane, (G) recovered input image Mandrill, and (H) recovered input image Peppers.

**Fig 5:**
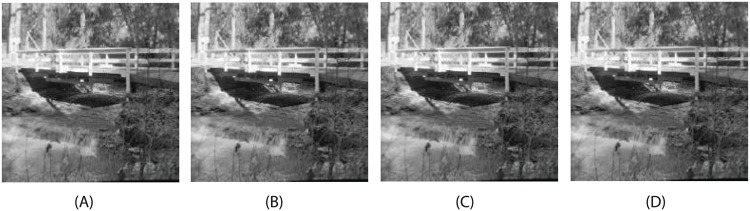
Extracted secret data (Bridge). (A) Extracted secret data from embedded image Lena, (B) extracted secret data from embedded image Airplane, (C) extracted secret data from embedded image Mandrill, and (D) extracted secret data from embedded image Peppers.

**Fig 6:**
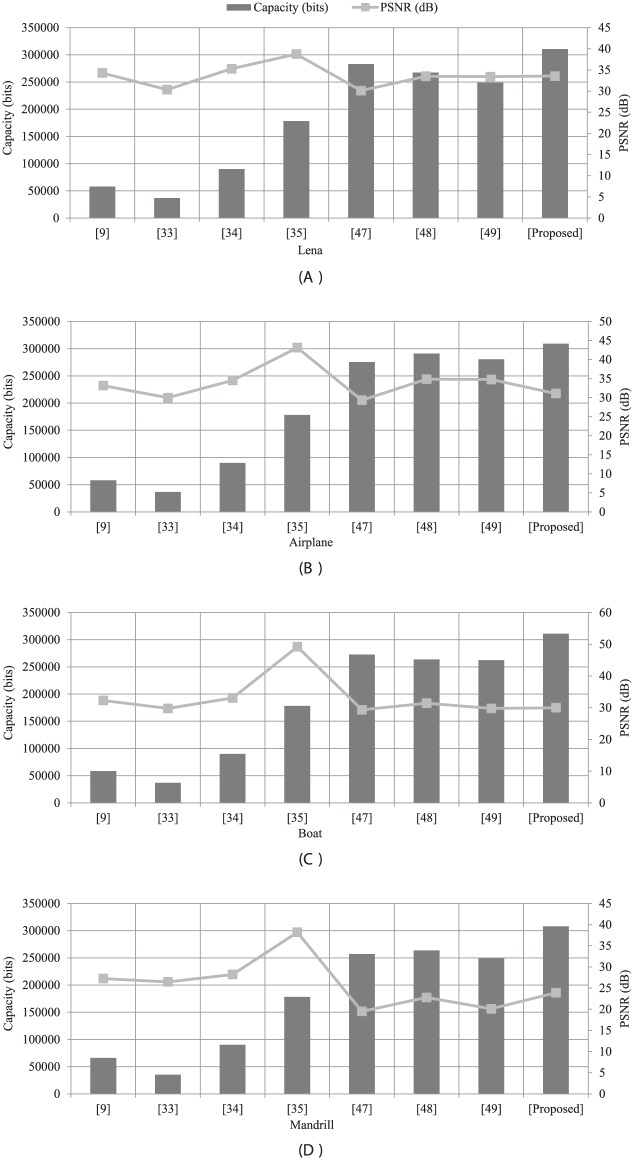
Graphical demonstration of the comparison of Table 4. (A) Capacity carried by Lena, (B) capacity carried by Airplane, (C) capacity carried by Boat, and (D) capacity carried by Mandrill. (Images information is given in Data Availability Statement.)

The carrying capacity of the information embedded images is estimated in terms of payload using the measurement of criteria in terms of bits per pixel (bpp). In Fig 4A–4D, the quality of the information embedded images is estimated in terms of PSNR (dB) is measured from an input image and embedded image [23]. In Table 3, the simulation outcomes of the proposed method are shown the Payload (bpp) values of the secret data capacity in Lena, Airplane, Mandril, and Peppers, respectively (a set of benchmark images) embedded images for comparing our proposed method with the watermarking method [24] and the interpolation based data hiding method [25]. In Fig 4E–4H, the lossless recovery of the input images are obtained with high imperceptibility. The corresponding extraction of secret images from embedded images Fig 4E–4H are shown with high accuracy in Fig 5A–5D. We empirically evaluate the choice of the strength factor based on the numerical simulation. Since the different values of *α* is acting as a weighting strength factor for the secret data, within the range of 0 to 1 are regulating the quality of the embedded image and value of the Payloads (bpp) of the extracted data. Note that with an increase in the value of the strength factor, the Payloads of the extracted data is increased and PNSR of embedded images is decreased and vice versa. In terms of payload, Table 3 shows that the proposed method is performed better than the [25], significantly. The scheme [25] was specially designed for interpolation based data hiding purpose. On the other hand, the method [24] was developed for watermarking purpose that carried only small amount of information in terms of ownership authentication [23]. Graphical demonstrations of the proposed method in terms of the embedding capacity with recent existing techniques are shown in Fig 6. Moreover, the proposed method delivers the improved imperceptibility of the embedded data along with large capacity values (the capacity size in bits [7]) as compared with recent existing methods as revealed in Table 4 and Fig 6. Note that the from Table 4 and Fig 6, it is clear that the proposed method offers higher embedding capacity than other existing methods. In Tables 3 and 4, there is a main reason for the higher PSNR values of the embedded image for the references [24, 28] as compare to our proposed method. Those methods [24, 28] are based on the non-blind data hiding schemes, where the information of the original cover image is demanded to be required to extract the embedded data, accurately. Note that in a non-blind data hiding method, embedding process can be optimally designed, so does to maintain the good quality of the information embedded data at the cost of less-capacity for embedding the secret data. On the other hand, in a blind data hiding method, embedding process is defined in such a way, so does to retrieve the embedded information without using of any clue from the original or cover image. However, in this case, the information embedded data has fairly lower quality as compared with the non-blind data hiding method. Furthermore, in our proposed method, due to the diffusion process of the Fresnelet transform application to the secret data, we can generate an encrypted data with coded pattern which has almost uniformly scattered structure, as discussed in [9]. This effect is justified in Fig 2. This pattern which would be embedded to a cover image is useful for keeping higher embedding capacity of secret data.

**Table 3:** Performance comparisons of the PSNR and Payload in [24], [25] and the proposed method.

Image	Sachnev et al. [24]		Jung and Yoo [25]		Proposed Method	
	PSNR	Payload	PSNR	Payload	PSNR	Payload
Lena	**58.18**	0.04	22.07	0.66	33.56	**1.185**
Airplane	**60.38**	0.04	22.62	0.70	31.07	**1.180**
Mandrill	**54.15**	0.04	20.09	**1.45**	23.89	1.175
Peppers	**55.55**	0.04	22.20	0.62	31.33	**1.189**

**Table 4:** Comparison of the quality of embedded images and the capacity of embedded secret data.

Image	Lena		Airplane		Boat		Mandrill	
Methods	Capacity	PSNR	Capacity	PSNR	Capacity	PSNR	Capacity	PSNR
[26]	36850	30.34	36817	29.98	36710	29.75	35402	26.46
[29]	57717	34.30	57966	33.16	56981	32.26	66075	27.27
[28]	90112	**35.28**	90112	34.53	90112	**33.05**	90112	**28.22**
[30]	283115	30.12	275251	29.33	272629	29.32	256901	19.51
[31]	267386	33.50	290979	34.86	263601	31.42	263601	22.79
[32]	249036	33.42	280494	**34.77**	262144	29.75	249036	20.10
Proposed	**310520**	33.56	**309216**	31.07	**31108**	29.97	**308032**	23.89

While, for most of other existing techniques [24, 25, 32], the secret data with low-capacity is considered to be hidden in a cover image without any diffusion process as shown in Tables 3, 4 and Fig 6, respectively. Ever since it is not possible to achieve the robustness or imperceptiveness of the secret embedded data and the capacity of the secret image simultaneously. However, to maximize, an adequate balance of these features would be prepared for a specific application [24, 25, 29]. For example, a secret data hiding method would forgo the robustness in favor of the higher capacity and low imperceptibility. On the other hand, an invisible watermarking method may not require large capacity of a watermark, would certainly favor of the higher imperceptibility in terms of the robustness of the cover image [24], which is demonstrated in Fig 6.

## Conclusion

In this work, a blind data hiding with twofold contributions is presented for secret data to handle substantially reversible approach and preserve latent detail information of retrieved-secret-data at extraction phase. At first stage, Fresnelet transform is employed for encrypting the secret data in the form of dummy complex data to be embedded in the cover image. Then the secret data and the original image were retrieved blindly and reversibly in the retrieving phase without the original cover image. Therefore, the proposed framework benefits from Fresnelet transform multi-resolution properties. Its performance in terms of quantitative quality was compared with recent state-of-the-art methods. The experimental results substantiate that it performs better than the listed algorithms and maintains excellent capacity for secret data hiding and can be used for achieving reasonable perceptual transparency of an image data. Towards this end, an anticipate to develop reversible data hiding algorithm for video encryption with desired improvements will be the subject of a future study.

## Supporting information

S1 File This is the appendix file. (PDF)
