# Supplementary material for: Reversible integer wavelet transform for blind image hiding method
Source: PLoS One. 2017 May 12;12(5):e0176979. doi: 10.1371/journal.pone.0176979 (PMC5428981; doi:10.1371/journal.pone.0176979)
Supplement: S1 File — (PDF) [file pone.0176979.s001.pdf]

S1 Appendix: Reversible Integer Wavelet Transform

December 17, 2016

Integer wavelet transforms (IWT) are invertible in finite-precision arithmetic, map integers to integers, and approximate linear wavelet transforms. Due largely to these properties, reversible IWT wavelet transforms are extremely useful for supporting functionalities such as the lossless reproduction of an original image [1, 2]. Often the invertibility of a transform depends on the fact that the transform is calculated using exact arithmetic [1] which demonstrates its finite-precision and invertible nature. In such a practice, however, an [1, 3, 4] arithmetic is inherently imprecise due to its limitation for rounding error scenario [1].

Polyphase of Signals and Filters

A fundamental tool in the study of multirate systems is the polyphase representation of a signal. Such a representation is defined with respect to a particular sampling matrix and corresponding set of coset vectors, and serves to decompose signal into a sum of signals called polyphase components [1].

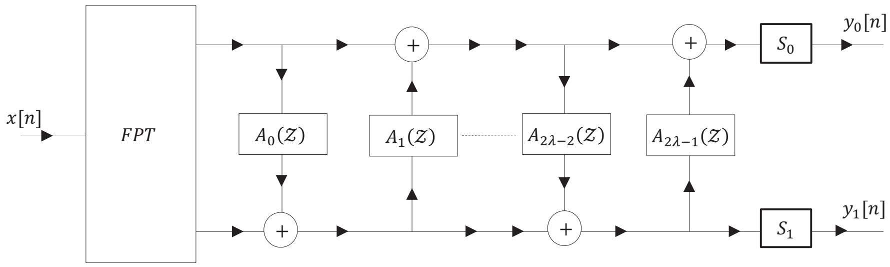

Figure 1: **The realizations of the integer wavelet transform via lifting scheme: the networks forward transform.**

Ladder Network

The ladder network yields symmetrical scaling and wavelet filter coefficients that have a linear phase. This linear phase property is made to use in the lifting algorithm which can be implemented using integer arithmetics that require only summations and register shifts [1, 2]. The ladder and its inverse step is demonstrated in Fig. 3 and Fig. 4.

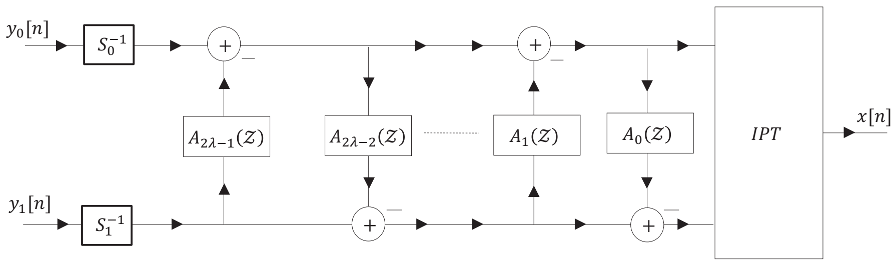

Figure 2: **The realizations of the integer wavelet transform via lifting scheme: the networks inverse transform.**

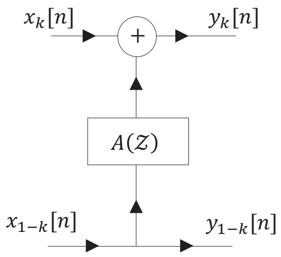

Figure 3: **The realizations of the integer wavelet transform via lifting scheme: the networks transform, a ladder step.**

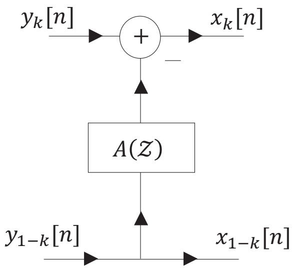

Figure 4: **The realizations of the integer wavelet transform via lifting scheme: the networks transform, a ladder inverse step.**

In proposed method embedding and retrieving processes are performed in WT domain. Recall that the integer wavelet transform is mapped integers to integers . Arithmetically, it has also finite-precision and invertible in nature. In such a practice, however, an arithmetic [1, 3, 4] is inherently imprecise due to its limitation for rounding error scenario [1]. In some technical sense, its invertibility depends on the exact computing of the arithmetic [1]. Fortunately, the integer WT is known as a perfect invertible transform using multirate lifting scheme. In the lifting scheme, it is particularly useful for supporting the lossless reproduction of an original image [1]-[3, 4].

Reversible Lifting Framework

General frameworks for reversible IWT wavelet transforms are most recent innovation. Note that, the lifting scheme for WT is realized by its filter banks known as Uniformly Maximally Decimated (UMD). These filter banks are carrying out the ladder networks to execute the polyphase filtering [1, 5]. Even in the existence of quantization error such networks can be acted invertible, especially, the rounding error is generated by using the finite-precision arithmetic. The process for realization of the WT via lifting scheme is described in Fig. 1 and Fig. 2. Let takes an input signal $x[n]$ and produces the output signal $y(n)$ and n is a D-dimensional integer column vector, where FPT and IPT denote forward and inverse polyphase transforms, respectively), as shown in Fig. 1 and Fig. 2. We can see that the polyphase filtering is performed by a ladder network and some additional amplifiers. The each filters in lifting-step constitutes a filter A_k, which is named as lifting-step filters.

The gains S_k are associated with the amplifiers, what is named a scaling step [6, 7]. Fig. 3 and Fig. 4 show two essential ladder steps of the lifting schemes. Specifically, Fig. 4 shows that the synthesis polyphase filtering $A(\mathcal{Z})$ cancels the polyphase filtering analysis effects in Fig. 3. Notice that the filter bank yielding the no shifting, with respect to original input signal. The filter bank is used to split and recombine a given data into its polyphase components [1].

In particular, the lifting scheme has been shown to provide a means for constructing reversible IWT wavelet transforms. Essentially, the lifting scheme is a polyphase realization strategy for handling the rounding error introduced by finite-precision arithmetic. To see why this is so, consider the two ladder steps shown in Fig. 3. Clearly, if exact arithmetic is employed, these two networks invert one another. Suppose now that the filtering operations with $A(Z)$ are implemented using finite-precision arithmetic and some round off error is incurred due to embedding of data. Since the two filters are identical (and assumed to use the same implementation strategy), they will both incur the same rounding error and their outputs will be identical. Therefore, whatever values are added in high frequency coefficients by the adder in the first network will be subtracted by the adder in the second network. Consequently, the two networks continue to invert one another, even in the presence of rounding error. This shows that ladder steps can maintain the invertibility of approximated data even in the presence of rounding error and we can reconstruct the distortion less original image as shown in Fig. 4. In other words, such networks are fundamentally reversible in nature. The proposed method used an integer version of the lifting up version of the Haar transform.

References

[1] Adams MD. Reversible integer-to-integer wavelet transforms for image coding: University of British Columbia; 2002.

[2] Sweldens W. The lifting scheme: A construction of second generation wavelets. SIAM Journal on Mathematical Analysis. 1998;29(2):511–46.

[3] Chang C-C, Lin C-C, Tseng C-S, Tai W-L. Reversible hiding in DCT-based compressed images. Information Sciences. 2007;177(13):2768–86.

[4] Peng F, Li X, Yang B. Adaptive reversible data hiding scheme based on integer transform. Signal Processing. 2012;92(1):54–62.

[5] Olkkonen H, Olkkonen J, Pesola P. Efficient lifting wavelet transform for microprocessor and VLSI applications. Signal Processing Letters, IEEE. 2005;12(2):120–2.

[6] Bruekers FAML, van den Enden AW. New networks for perfect inversion and perfect reconstruction. Selected Areas in Communications, IEEE Journal on. 1992;10(1):129–37.

[7] Shah IA, Kalker TA, editors. On ladder structures and linear phase conditions for bi-orthogonal filter banks. Acoustics, Speech, and Signal Processing, 1994 ICASSP-94, 1994 IEEE International Conference.
